# Decreased diabetes risk over 9 year after 18-month oral l-arginine treatment in middle-aged subjects with impaired glucose tolerance and metabolic syndrome (extension evaluation of l-arginine study)

**DOI:** 10.1007/s00394-017-1548-2

**Published:** 2017-10-20

**Authors:** Lucilla D. Monti, Elena Galluccio, Valentina Villa, Barbara Fontana, Serena Spadoni, Pier Marco Piatti

**Affiliations:** 1 Cardio-Diabetes and Core Lab Unit, Department of Internal Medicine, Diabetes Research Institute, San Raffaele Scientific Institute, Via Olgettina 60, 20132 Milan, Italy; 2 Cardio-Metabolism and Clinical Trials Unit, Department of Internal Medicine, Diabetic Research Institute, San Raffaele Scientific Institute, Milan, Italy

**Keywords:** l-Arginine, Endothelial function, Insulin secretion, Prevention of type 2 diabetes, Oxidative stress

## Abstract

**Purpose:**

This study aimed to determine whether l-arginine supplementation lasting for 18 months maintained long-lasting effects on diabetes incidence, insulin secretion and sensitivity, oxidative stress, and endothelial function during 108 months among subjects at high risk of developing type 2 diabetes.

**Methods:**

One hundred and forty-four middle-aged subjects with impaired glucose tolerance and metabolic syndrome were randomized in 2006 to an l-arginine supplementation (6.4 g orally/day) or placebo therapy lasting 18 months. This period was followed by a 90-month follow-up. The primary outcome was a diagnosis of diabetes during the 108 month study period. Secondary outcomes included changes in insulin secretion (proinsulin/c-peptide ratio), insulin sensitivity (IGI/HOMA-IR), oxidative stress (AOPPs), and vascular function. After the 18 month participation, subjects that were still free of diabetes and willing to continue their participation (104 subjects) were further followed until diabetes diagnosis, with a time span of about 9 years from baseline.

**Results:**

Although results derived from the 18 month of the intervention study demonstrated no differences in the probability of becoming diabetics, at the end of the study, the cumulative incidence of diabetes was of 40.6% in the l-arginine group and of 57.4% in the placebo group. The adjusted HR for diabetes (l-arginine vs. placebo) was 0.66; 95% CI 0.48, 0.91; *p* < 0.02). Proinsulin/c-peptide ratio (*p* < 0.001), IGI/HOMA-IR (*p* < 0.01), and AOPP (*p* < 0.05) levels were ameliorated in l-arginine compared to placebo.

**Conclusions:**

These results may suggest that the administration of l-arginine could delay the development of T2DM for a long period. This effect could be mediated, in some extent, by l-arginine-induced reduction in oxidative stress.

## Introduction

Worldwide, the incidence of type 2 diabetes mellitus (T2DM) is increasing. All regions are projected to have an increase in the numbers of people with diabetes larger than those for growth in the adult population alone. Moreover, prediabetes represents a high-risk state for the development of type 2 diabetes (T2DM) and cardiovascular disease must be considered itself a disease [1–3]. In prediabetes, β-cell volume is already lost for 30–40% and this alteration is associated with a progression of insulin resistance to increased insulin demand [4]. Disproportionately elevated intact proinsulin levels in the peripheral blood serve as an appropriate laboratory marker for this phenomenon by disclosing the exhausted cleavage capacity of intra-cellular processing enzymes [5]. During this period, pancreatic β-cells are also vulnerable to oxidative stress as a consequence of either excessive reactive oxygen species (ROS) production or a failure of antioxidant species to effectively neutralize increasing ROS levels. This determines an alteration in intra-cellular signaling, β-cell destruction, and dysfunction [6, 7]. ROS have been shown to increase also protein arginine methyltransferase and inhibit dimethylarginine dimethylaminohydrolase activity, leading to an increase in asymmetric dimethyl arginine (ADMA) levels [8]. Furthermore, high levels of ADMA can uncouple NOS isoenzymes to produce superoxide instead of NO, contributing to burden oxidative stress [9]. Moreover, oxidative stress can impair endothelial function and decreases number and function of endothelial progenitor cells (EPCs) [10, 11].

Some recent studies have demonstrated beneficial effects of the use of l-arginine supplementation as a nutrient treatment in diabetes and prediabetes. l-Arginine, is a conditionally essential amino acid, and is found commonly in many foods. The most common sources of arginine are meat, poultry, fish, dairy products, and plant sources (fruits, vegetables, nuts, legumes, and grains) [12]. Results obtained from the NHANES III showed that the median daily arginine intake is 3.8 g/day [13].

l-Arginine is an amino acid involved in various metabolic pathways and it is a substrate for the family of NOS enzymes that generate nitric oxide, a key molecule involved in normal endothelial function and insulin sensitivity [14, 15] as well as metabolic profile [16], particularly in subjects at risk of developing type 2 diabetes [17–20]. Since the previous studies have found that l-arginine is also able to improve endothelial [17–20], β-cell function [21, 22], and oxidative stress [23], the aim of this study was to investigate whether l-arginine may influence the incidence of new diabetic diagnosis, improving endothelial function, β-cell function, insulin sensitivity, and oxidative stress after 9 years from the initiation of an oral 18 month treatment with l-arginine in people at high risk for T2DM development.

## Research design and methods

### Study design and patient population

**Fig. 1:**
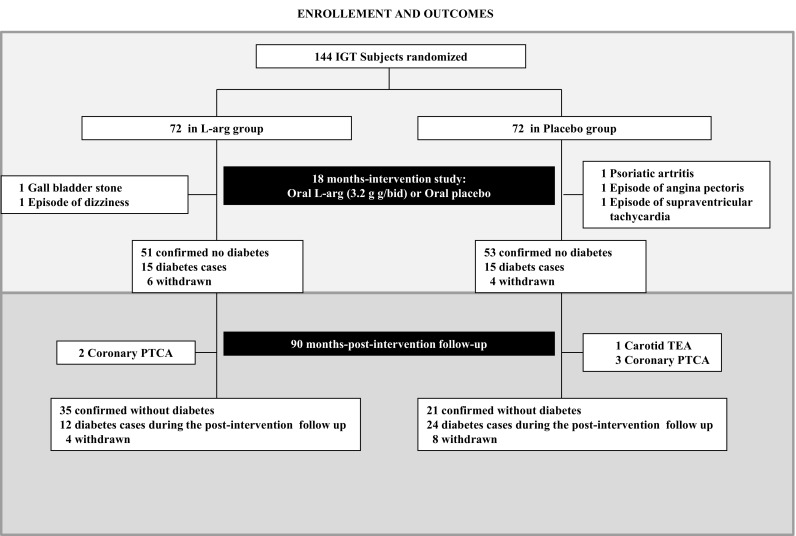
Trial profile. One hundred and forty-four subjects were randomized to the active arm (72 patients) or to the placebo arm (72 subjects). One hundred and thirty-four subjects (66 in l-arginine group and 68 in the placebo group) completed the treatment period (104 confirmed without diabetes, 30 cases diagnosed for diabetes, and 10 withdrawn). The 104 subjects confirmed without diabetes entered the 90-month postintervention follow-up. Of those, 92 subjects (47 and 45 subjects, respectively) completed the follow-up period with 12 subjects being withdrawn

One hundred and forty-four middle-aged subjects with impaired glucose tolerance and metabolic syndrome were randomized in 2006 to an l-arginine supplementation (6.4 g orally/day) or placebo therapy lasting 18 months, and all randomized patients were included in the analyses [20]. Subjects were censored from study on the day of their follow-up visit/withdrawal date, since becoming diabetics and results occurring after follow-up visit were not included. Thus, after 18-month study, subjects still free of diabetes and willing to continue entered in the follow-up study (104 subjects) where they were further followed until the end of 2014 or at diabetes diagnosis or when becoming a drop out. Results were taken after a time span of 9 years from baseline. Trial profile that includes either 18-month intervention period or 90-month follow-up post intervention period is reported in Fig. 1. At the end of the intervention period, in the l-arginine group, in 51 subjects, the absence of diabetes was confirmed (23 subjects remained IGT and 28 subjects became NGT), in 15 subjects, the presence of diabetes was defined and 6 subjects were withdrawn. In the placebo group, in 53 subjects, the absence of diabetes was confirmed (38 subjects remained IGT and 15 subjects became NGT, *p* < 001 vs. arginine group); in 15 subjects, the presence of diabetes was defined and 4 subjects were withdrawn. Ninety-two subjects (47 subjects in l-arginine group and 45 subjects in placebo group, respectively) completed the 90-month follow-up period.

The primary outcome was the diagnosis of diabetes based on OGTTs performed every 6 months. Secondary outcomes included changes in insulin secretion (proinsulin/c-peptide ratio), insulin sensitivity (IGI/HOMA-IR), oxidative stress by the measurement of advanced oxidation protein products (AOPPs), and vascular function. The inclusion and exclusion criteria and the results of the 18-month intervention study are reported in a previously published manuscript [20].

The study and all procedures performed were approved by the local Ethic Committee of the San Raffaele Scientific Institute, and conducted in accordance with the International Conference on Harmonisation Guidelines on Good Clinical Practice and the principles of the 1964 Declaration of Helsinki and its later amendments or comparable ethical standards. All patients provided written informed consent. The study is registered with ClinicalTrials.gov, number NCT 00917449 and EudraCT, number 2005-004639-24.

### Methods to measure insulin sensitivity and secretion and endothelial function

During the annual visit, each subject performed a clinical and anthropometric evaluation and a 75-g oral glucose tolerance test (OGTT) was performed. The OGTT was used to define glucose tolerance and to evaluate insulin secretion and sensitivity during post prandial (postglucose load) period. Blood samples were drawn during the OGTT for the measurement of plasma glucose, serum insulin, serum proinsulin, serum c-peptide, proinsulin/c-peptide ratio, and lipid levels. Serum insulin levels were assayed with ELISA KIT (Mercodia, Uppsala, Sweden) with a sensitivity of 1 µU/ml and intra- and inter-assay coefficients of variation (CVs) of 3.0 and 5.0%, respectively. Human proinsulin (ELISA, DRG, Marburg, Germany) was assayed with no cross reactivity with human insulin. The minimum detectable dose was 6 pmol/l. Intra-assay CV was 3.5% and inter-assay CV was 5.5%. Serum C-peptide levels was assayed with an ELISA KIT (Mercodia, Uppsala, Sweden) with intra-assay CV 3.0% and inter-assay CV 4.0%.

Fasting insulin resistance was measured by the homeostasis model of assessment of insulin resistance (HOMA-IR) using the formula HOMA-IR = (fasting insulin × fasting glucose)/22.5, as described by Matthews and colleagues [24].

The insulinogenic index (IGI), with IGI defined as the incremental change in insulin during the first 30 min of the OGTT divided by the incremental change in blood glucose over the same period [(30 min insulin-fasting insulin)/(30 min glucose-fasting glucose)].

To account for the compensatory response of insulin secretion in relation to background insulin resistance (i.e., disposition index), IGI was divided by the HOMA-IR to yield IGI/HOMA-IR [25–27].

Fasting proinsulin levels and proinsulin/c-peptide ratio were used to evaluate β-cell function.

AOPP, NOx, asymmetric dimethylarginine (ADMA), and endothelial progenitor cells-colony forming units (CFU-EPCs) were evaluated at baseline, at the end of the intervention study, and during the post intervention period at 48 months and at 108 months.

AOPP was assayed on EDTA plasma with an Elisa kit following the manufacturer’s instructions (Immundiagnostik AG, Stubenwald-Allee 8a, D-64625 Bensheim, Germany).

For the measurement of vascular function, the number of CFU-EPCs was measured as described by Hill et al. [28]. Peripheral blood mononuclear cells were isolated by Ficoll density gradient centrifugation. Recovered cells were suspended in a growth medium (Medium 199 supplemented with 20% fetal bovine serum, 100 U/ml penicillin, 100 μg/ml streptomycin; Sigma-Aldrich, St. Louis, MO, USA) and plated on endothelial cell attachment factor-coated dishes (Sigma-Aldrich, St. Louis, MO USA) for 48 h. Non-adherent cells were then recollected, replated onto endothelial cell attachment factor-coated 24-well plates at a density of 1 × 10^6^ cells/well, and cultured for 7 days in a growth medium that was changed every 3 days. CFUs, which were characterized by a central cluster surrounded by emerging cells, were then counted.

ADMA was assayed on EDTA plasma with an Elisa kit following the manufacturer’s instructions (DLD Diagnostika GmbH, Adlerhorst 15 D-22459 Hamburg, Germany) with a sensitivity of 0.05 mmol/l; within-assay variation was 5.7%, and between-assay variation was 9.8%.

### Methods to evaluate dietary intake and physical activity

All subjects received a lifestyle intervention consisting of a one-on-one session lasting about 30 min during each visit throughout the study, scheduled every 3 months in the treatment period. During each session, all subjects were given general oral and written information about diet (1600 kcal/day with 55% carbohydrate, 25–30% fat and 15–20% protein) and exercise. During the intervention study and during the postintervention follow-up period, they completed a 3-day food diary at baseline, and at each visit, using a booklet illustrating the sizes of portions of food. Food diaries were processed using dedicated software (Nutritionist Pro 2.5, Axial System, Stafford, TX, USA) modified by introducing the l-arginine content obtained from the INRAN and USDA databases for more than 700 different food items. Self-reported levels of leisure physical activity were assessed at baseline, at the end of the interventional trial period, and at the end of the follow-up period with the Modifiable Activity Questionnaire [29]. The physical activity level was calculated as the product of the duration and frequency of each activity in hours per week.

### Statistical analysis

Baseline characteristics of the two study groups were summarized with means and standard deviations for continuous variables, and frequencies and percentages for categorical variables.

Kaplan–Meier survival curves were calculated to estimate the probability of remaining free of diabetes in the two groups.

The difference between the survival curves was tested using the log-rank test. The Cox proportional hazard model was used to estimate the HR for the development of diabetes. Cox proportional hazard model was also used to assess the effect of l-arginine and placebo on the hazard of the time to regression to NGT.

All variables were compared between the groups of subjects that remained free of diabetes throughout the study period using repeated-measures analysis of variance (ANCOVA analysis). The Wilcoxon rank-sum test was used to assess differences between the two study groups for continuous variables (proinsulin, proinsulin/c-peptide ratio, IGI, HOMA, IGI/HOMA-IR ratio, ADMA, AOPP ,and EPC) up to 108-month follow-up period.

The *χ* ^2^ test (or Fisher’s exact test when necessary) and the median test were performed in these subjects for categorical variables. All analyses were done with SPSS (version 15.0). A *p* value less than 0.05 was reported as statistically significant.

The sample size has been estimated taking advantage from a previous intervention study performed in a small number of subjects affected by CAD with IGT [19] in which the main objective was the improvement in insulin sensitivity after 6 month of supplement of l-arginine (6.4 g, 16 subjects) vs. placebo (14 subjects) associated with changes in life style. We found that at the end of this period, the incidence of diabetes was reduced by 70% with l-arginine as compared to placebo (1/14 subjects vs. 4/16 subjects, respectively). Moreover, in the l-arginine group, seven subjects returned to be normal glucose tolerant after OGTT vs. only two subjects with placebo. Also taking into account other studies evaluating the median time for the development of type 2 diabetes, from epidemiological and intervention studies performed in patients with IGT [30–32], we calculated an annual incidence of IGT subjects of 6.5%/year. Taking into consideration these values, we calculated that a sample size of 70 subjects per study group would provide a relative risk of 0.50, with a two-sided log-rank test at a significance level of 0.05. This calculation included a drop-out rate of 1.5–1.7%/year.

## Results

### Probability of remaining free of diabetes or to remained NGT

**Fig. 2:**
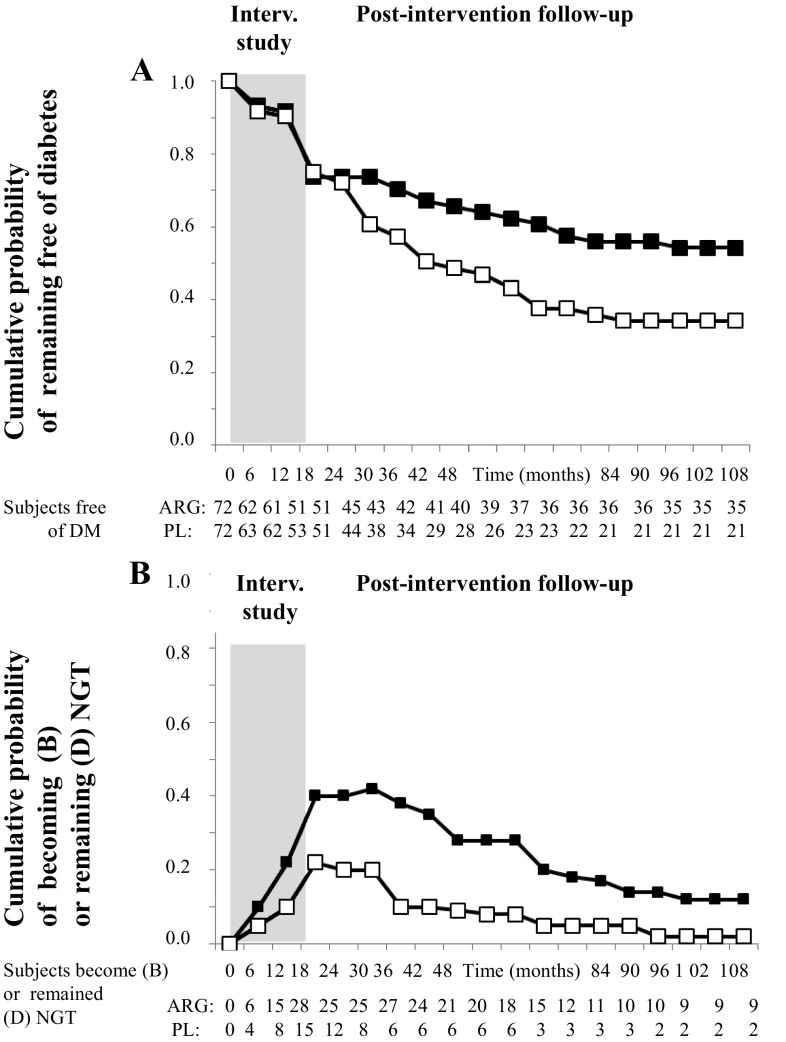
Kaplan–Meier estimates cumulative probability of remaining free of diabetes and of becoming/remaining NGT. The 2-h postload plasma glucose levels were measured at baseline and every 6 months. The outcomes were evaluated at the end of the study. **a** Total follow-up of cumulative probability of remaining free of diabetes in l-arginine (black boxes) and placebo groups (white boxes). As compared to placebo group, the HR was 0.66 (95% CI 0.48, 0.91; *p* < 0.05) in the l-arginine group. **b** Total follow-up of cumulative probability of becoming/remaining NGT in l-arginine (black boxes) and placebo groups (white boxes). As compared to placebo group, the HR was 1.38 (95% CI 0.99, 1.93; *p* = 0.33) in l-arginine group

Figure 2 reports the Kaplan–Meier survival curves to estimate the probability of remaining free of diabetes and the probability of becoming/remaining NGT.

As previously reported [18], results derived from the 18 month intervention study demonstrated that there was not differences in the probability of becoming diabetics between l-arginine or placebo, being 21.4% of subjects in the l-arginine group and 20.8% of participants in the placebo group (HR 1.04; 95% CI 0.58–1.86; *p* = 0.91). In particular, in the l-arginine group during the intervention study, 15 subjects became diabetics and 51 subjects remained free of diabetes, while, in the postintervention period, 12 subjects became diabetics and 35 remained free of diabetes. Conversely, in the placebo group during the intervention study, 15 subjects became diabetics and 53 subjects remained free of diabetes, while in the postintervention period, 24 subjects became diabetics and only 21 remained free of diabetes.

Therefore, at the end of the study, 27 and 39 subjects after l-arginine and after placebo became diabetics (Fig. 1). At the end of the study, the cumulative incidence of diabetes was of 40.6% in the l-arginine group, and of 57.4% in the placebo group (HR 0.66; 95% CI 0.48, 0.92; *p* < 0.05) (Fig. 2a). Diabetes incidence rates per 100 person-years were 3.0/year (5%/year) in the l-arginine group and 4.2/year (6.9%/year) in the placebo group.

Whereas at the end of the intervention study period, 28 subjects (42.4%) receiving l-arginine, compared with 15 subjects (22.1%) receiving placebo returned to NGT (HR 2.60; 95% CI 1.51–4.46; *p* = 0.001), at the end of the postintervention follow-up, only 9 subjects in l-arginine and 2 subjects in placebo remained NGT with an adjusted HR of 1.38 (95% CI 0.99, 1.93; *p* = 0.33) (Fig. 2b). Even if the analysis included a small number of subjects, the median period to remain NGT after the intervention study was almost doubled with l-arginine as compared to placebo (72 vs. 33 months, respectively; *p* < 0.05).

### Profiles of metabolic, insulin secretion, and insulin sensitivity, and endothelial and oxidative stress variables

All variables were compared between the groups of subjects that remained free of diabetes throughout the study period (35 and in 21 subjects in the l-arginine and placebo groups, respectively).

**Fig. 3:**
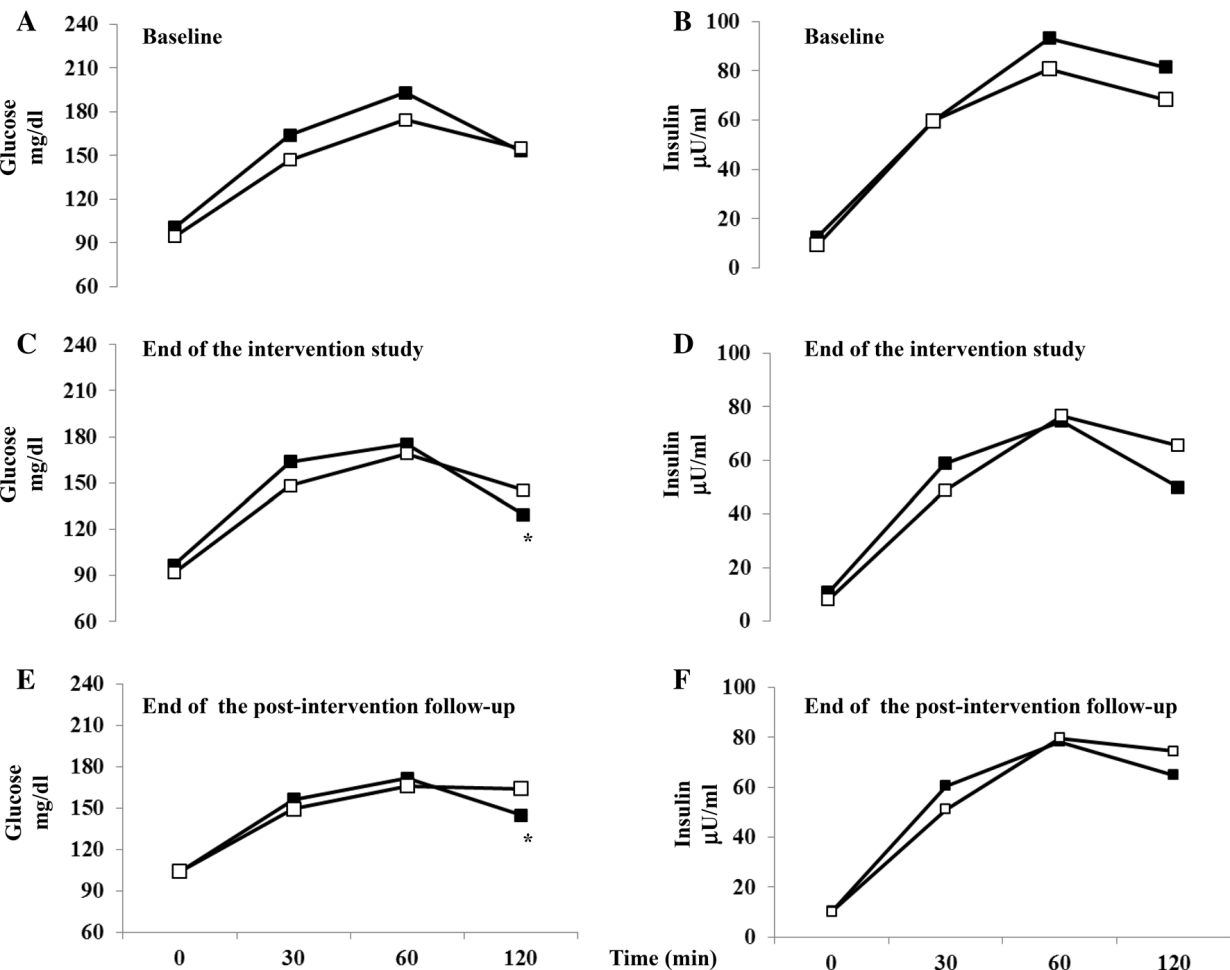
Mean glucose (**a**, **c**, **e**) and insulin (**b**, **d**, **f**) levels at baseline, at the end of the intervention study and at the end of the postintervention follow-up in l-arginine (black boxes) and in placebo (white boxes) groups during OGTT. *p < 0.05 vs. placebo

In Fig. 3, mean glucose and insulin levels during OGTT are represented at baseline (Fig. 3a, b), at the end of the intervention study (Fig. 3c, d) and at the end of the postintervention study. While at baseline, glucose and insulin levels were similar in both groups, glucose levels significantly decreased by 10.8% in l-arginine group as compared to placebo group at the end of the intervention study (at 120 min: 129.5 ± 33.3 vs. 145.3 ± 27.3 mg/dl, *p* < 0.05) and by 11.7% at the end of the post intervention period (at 120 min: 145.0 ± 40.0 vs. 164.3 ± 48.7 mg/dl, *p* < 0.05). Similar improvement was also found measuring insulin levels, although this improvement did not reach statistical differences.

**Fig. 4:**
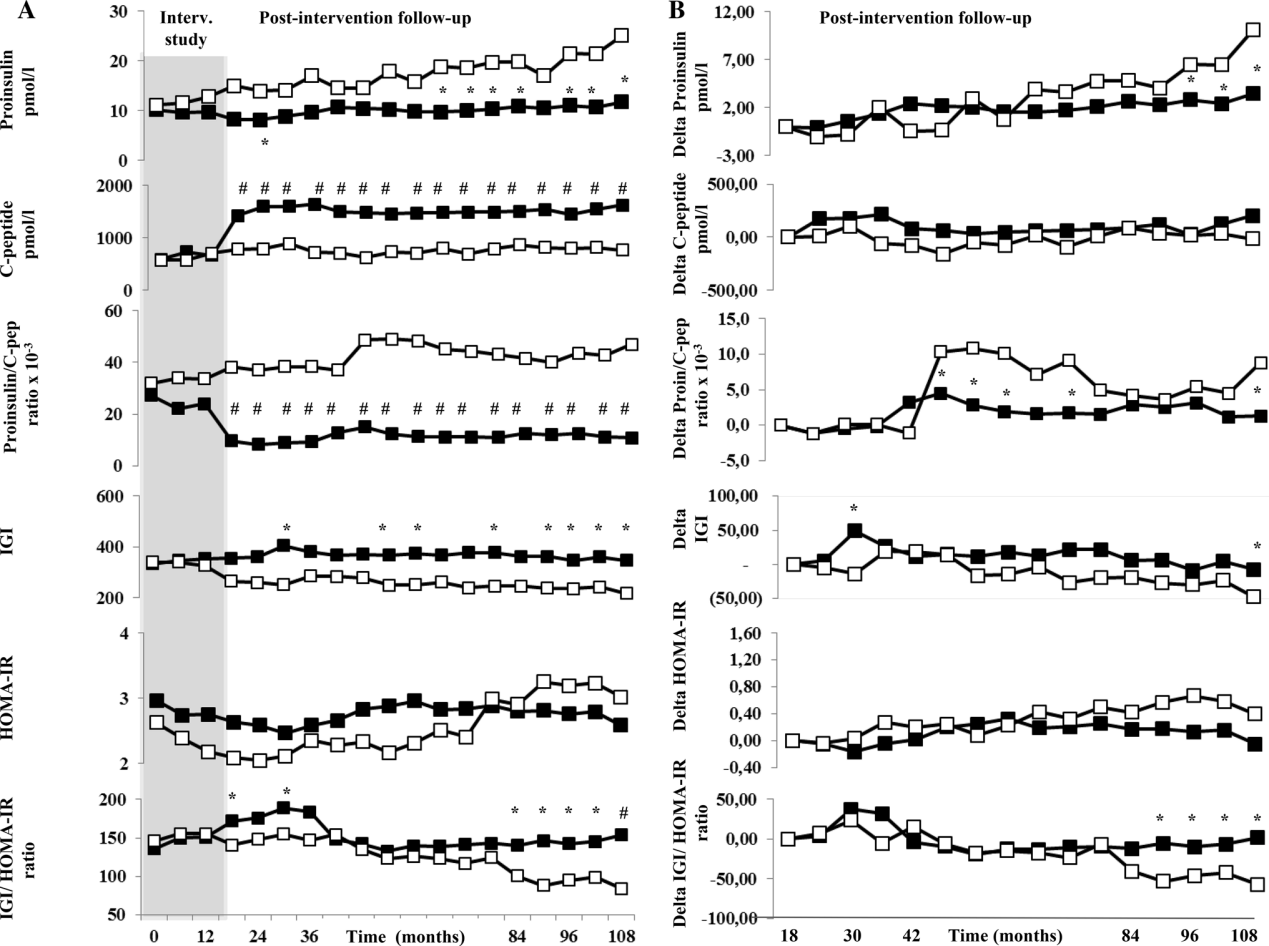
**a** Mean fasting proinsulin, proinsulin/c-peptide ratio, IGI, HOMA-IR, and IGI/HOMA-IR ratio in 35 and in 22 persons in the l-arginine (black boxes) and placebo (white boxes) groups, respectively, that remained free of diabetes at the end of the postintervention follow-up during the total period of study (intervention study and postintervention follow-up period). *p < 0.05 vs. placebo; ^#^ *p* < 0.01 vs. placebo. **b** Change difference during the postintervention period compared to results of each variable obtained at the end of the intervention study for fasting proinsulin, proinsulin/c-peptide ratio, and IGI HOMA-IR and IGI/HOMA-IR ratio in 35 and in 22 subjects in the l-arginine (black boxes) and placebo (white boxes) groups, respectively, that remained free of diabetes at the end of the postintervention follow-up period. *p < 0.05 vs. placebo; **p < 0.01 vs. placebo. *HOMA-IR* homeostasis model assessment-insulin resistance, *IGI* insulinogenic index

In Fig. 4a, indices of insulin secretion and insulin sensitivity in these subjects are represented during the total follow-up period of 9 years. All variables were similar at baseline. While mean proinsulin levels remained stable throughout the study period in l-arginine group, mean proinsulin levels in the placebo group significantly increased from baseline by 25.3% at the end of the intervention period and by 55.4% in the postintervention follow-up (at 108 months: l-arginine group: 11.7 ± 9.1 vs. placebo group: 25.1 ± 17.6 pmol/l, *p* < 0.05). Conversely, compared to placebo that remained stable throughout the study period, mean c-peptide levels increased by 41.8% in the l-arginine group at the end of the intervention period and remained almost stable throughout the postintervention follow-up period (at 108 months: 1628.6 ± 1400.3 vs. 769.5 ± 748.6 pmol/l, *p* < 0.01): Therefore, proinsulin/c-peptide ratio was fourfold higher in placebo group than in l-arginine group (at 108 months: 47.3 ± 46.6 vs. 11.2 ± 13.8 × 10^−3^, *p* < 0.01).

Compared to baseline, mean IGI levels remained almost stable in the l-arginine group, while IGI levels in the placebo group significantly decreased by 35.8% (at 108 months: 347.4 ± 164.4 vs. 217.9 ± 202.4, *p* < 0.05) suggesting a significant impairment in insulin secretion in placebo group during this period. Moreover, IGI/HOMA-IR ratio, variable that explores the disposition index, was almost twofold higher in the l-arginine group than in placebo group at the end of the study (at 108 months: 153.6 ± 90.6 vs. 83.8 ± 111.6, *p* < 0.05). The data strongly suggest that l-arginine preserved β-cells function and disposition index lasting 108 months of observation.

**Fig. 5:**
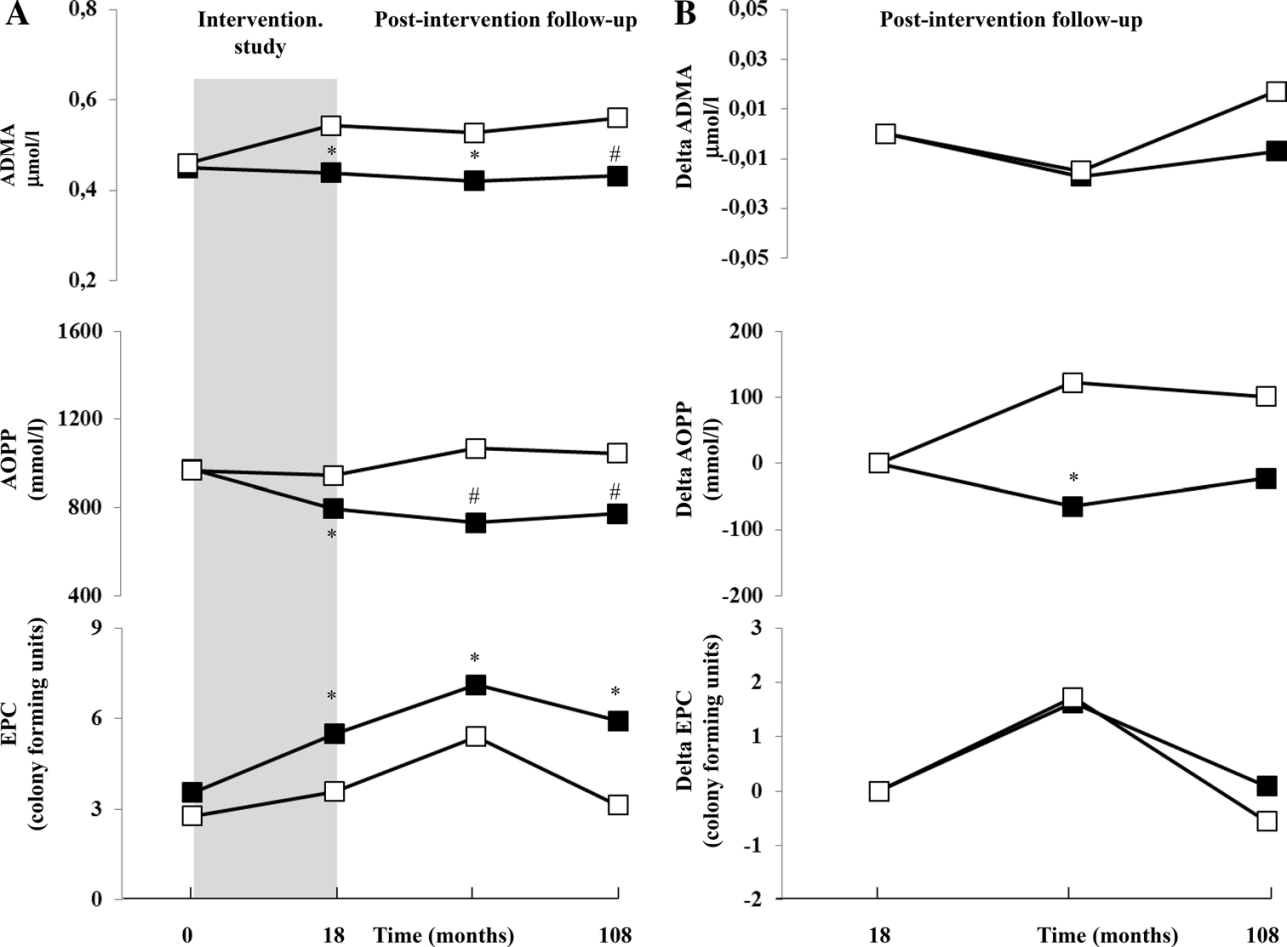
**a** Mean ADMA, AOPP, and EPCs levels in 35 and in 22 subjects in the l-arginine (black boxes) and placebo (white boxes) groups, respectively, that remained free of diabetes at the end of the postintervention follow-up during the total period of study (intervention study and postintervention follow-up period). *p < 0.05 vs. placebo; ^#^ *p* < 0.01 vs. placebo. **b** Change difference during the post intervention period compared to results of each variable obtained at the end of the intervention study for ADMA, AOPP, and EPCs in 35 and in 22 subjects in the l-arginine (black boxes) and placebo (white boxes) groups, respectively, that remained free of diabetes at the end of the postintervention follow-up period. *p < 0.05 vs. placebo; **p < 0.01 vs. placebo. *ADMA* asymmetric dimethylarginine, *AOPP* advanced oxidation protein products, *EPCs* endothelial progenitor cells

Profiles of indices of oxidative stress and endothelial function are represented in Fig. 5. All variables were similar at baseline. Compared to placebo AOPP and ADMA levels significantly decreased by 26.1% and by 23.2% at the end of the intervention period, remaining still lower during the post intervention period in l-arginine group (AOPP at 108 months: 773.2 ± 244.4 vs. 1045.7 ± 282.9 μmol/l, *p* < 0.05; ADMA at 108 months: 0.43 ± 0.14 vs. 0.56 ± 0.14 μmol/l, *p* < 0.05). Conversely, EPCs in l-arginine group were 44.8% higher than placebo group at the end of the intervention study (*p* < 0.05) and remained significantly higher during the postintervention period (at 108 months: 5.93 ± 1.37 vs. 3.56 ± 1.88 colony form units, *p* < 0.05). These data strongly suggest that l-arginine improves endothelial function and oxidative stress even after 108 months of observation.

The repeated-measures analysis of variance confirms that time for treatment analysis was significantly different between the two groups for glucose and insulin at 2-h postglucose load, IGI/HOMA-IR, proinsulin/c-peptide ratio, AOPP and ADMA levels, and EPC-CFU (Tables 1, 2).

**Table 1:** ANCOVA analysis of glucose and insulin levels, insulin sensitivity, and insulin secretion indices and endothelial function in 35 participants in the l-Arg group and 21 participants in the placebo group that remained non-diabetic at the end of the postintervention follow-up (mean ± SD)

Variable	Baseline	Intervention study	Postintervention follow-up	Time *p* value	Time for treatment *p* value
Fasting glucose (mg/dl)					
l-Arg	100.5 ± 12.3	96.5 ± 13.4	103.9 ± 13.5	0.12	0.55
Placebo	94.1 ± 12.5	91.5 ± 11.4	104.1 ± 13.3		
2-h-glucose (mg/dl)					
l-Arg	153.9 ± 13.4	129.5 ± 33.3	145.0 ± 40.0	0.39	0.05
Placebo	154.8 ± 12.0	145.3 ± 27.3	164.3 ± 48.7		
Fasting insulin (mU/ml)					
l-Arg	12.4 ± 5.5	10.6 ± 5.9	10.2 ± 6.0	0.65	0.51
Placebo	9.4 ± 7.7	8.0 ± 4.7	10.0 ± 5.3		
2-h-insulin (mU/ml)					
l-Arg	81.5 ± 56.8	49.8 ± 44.9	54.9 ± 39.6	0.60	0.05
Placebo	68.4 ± 49.9	65.6 ± 44.3	65.6 ± 41.1		
IGI					
l-Arg	333.6 ± 222.3	355.1 ± 237.6	347.4 ± 164.4	0.58	0.05
Placebo	339.4 ± 150.2	265.4 ± 180.7	217.9 ± 202.4		
HOMA-IR					
l-Arg	2.97 ± 1.44	2.64 ± 1.57	2.59 ± 1.64	0.58	0.42
Placebo	2.63 ± 1.79	2.10 ± 1.18	3.02 ± 1.58		
IGI/HOMA-IR					
l-Arg	143.6 ± 66.6	152.0 ± 13.8	153.6 ± 90.6	0.48	0.01
Placebo	150.9 ± 53.2	140.8 ± 96.2	83.8 ± 111.6		
Proinsulin (pmol/l)					
l-Arg	10.2 ± 7.3	8.3 ± 4.7	11.7 ± 9.1	0.44	0.01
Placebo	11.2 ± 12.5	15.0 ± 13.1	25.1 ± 17.6		
C-peptide (pmol/l)					
l-Arg	596.9 ± 636.5	1428.9 ± 831.7	1628.6 ± 1400.3	0.07	0.05
Placebo	579.6 ± 847.1	786.6 ± 653.9	769.5 ± 748.6		
Proinsulin/c-pep (×10^−3^)					
l-Arg	27.1 ± 35.3	10.2 ± 13.0	11.2 ± 13.8	0.89	0.001
Placebo	32.0 ± 22.1	38.4 ± 40.1	47.3 ± 46.6		
ADMA (mmol/l)					
l-Arg	0.45 ± 0.11	0.44 ± 0.15	0.43 ± 0.14	0.05	0.01
Placebo	0.46 ± 0.14	0.54 ± 0.17	0.56 ± 0.14		
EPC (colony forming unit)					
l-Arg	3.55 ± 1.95	5.50 ± 1.05	5.93 ± 1.37	0.12	0.05
Placebo	2.77 ± 1.71	3.68 ± 1.61	3.56 ± 1.88		
Nox (mmol/l)					
l-Arg	17.9 ± 12.9	12.9 ± 8.3	12.1 ± 10.4	0.48	0.16
Placebo	15.9 ± 9.0	15.0 ± 8.7	17.0 ± 16.9

**Table 2:** ANCOVA analysis of clinical characteristics, lipid levels, and oxidative stress in 35 participants in the l-Arg group and 21 participants in the placebo group that remained non-diabetic at the end of the postintervention follow-up (mean ± SD)

Variable	Baseline	Intervention study	Postintervention follow-up	Time *p* value	Time for treatment *p* value
Body weight (kg)					
l-Arg	88.2 ± 12.9	82.7 ± 13.1	81.8 ± 11.8	0.05	0.55
Placebo	82.8 ± 12.2	81.8 ± 11.8	83.9 ± 14.3		
Fat mass (kg)					
l-Arg	31.5 ± 10.9	26.3 ± 9.9	27.6 ± 10.7	0.08	0.15
Placebo	27.7 ± 8.2	26.4 ± 6.6	25.6 ± 8.6		
Fat free mass (kg)					
l-Arg	56.5 ± 7.6	55.7 ± 9.4	56.4 ± 10.2	0.65	0.05
Placebo	55.0 ± 7.6	51.1 ± 7.9	51.8 ± 8.1		
Waist circumference (cm)					
l-Arg	104.0 ± 9.9	99.2 ± 10.6	99.3 ± 10.3	0.08	0.05
Placebo	99.7 ± 9.9	97.3 ± 8.0	100.2 ± 8.0		
Systolic B. press (mmHg)					
l-Arg	129.3 ± 16.7	128.1 ± 20.3	126.0 ± 17.7	0.76	0.75
Placebo	123.6 ± 14.4	127.5 ± 14.5	123.2 ± 17.3		
Diastolic B. press (mmHg)					
l-Arg	79.6 ± 8.5	80.3 ± 8.8	79.1 ± 9.8	0.82	0.61
Placebo	77.5 ± 9.0	82.0 ± 9.1	79.1 ± 7.8		
Tot. cholesterol (mg/dl)					
l-Arg	176.4 ± 37.3	166.8 ± 34.5	173.9 ± 26.3	0.05	0.15
Placebo	196.8 ± 39.7	184.9 ± 37.7	175.7 ± 44.8		
HDL cholesterol (mg/dl)					
l-Arg	37.8 ± 10.3	41.7 ± 10.5	43.5 ± 12.3	0.05	0.17
Placebo	41.6 ± 11.4	43.7 ± 12.4	44.5 ± 13.9		
LDL cholesterol (mg/dl)					
l-Arg	111,4 ± 31.9	107.8 ± 33.4	110.8 ± 25.9	0.01	0.75
Placebo	125.4 ± 37.2	114.2 ± 35.4	104.4 ± 37.8		
Triglycerides (mg/dl)					
l-Arg	136.0 ± 70.8	86.9 ± 29.8	101.5 ± 39.0	0.05	0.57
Placebo	125.7 ± 99.9	110.1 ± 85.7	108.6 ± 73.5		
Free fatty acids (mmol/l)					
l-Arg	0.57 ± 0.22	0.65 ± 0.34	0.77 ± 0.49	0.05	0.53
Placebo	0.57 ± 0.22	0.77 ± 0.24	1.11 ± 0.72		
AOPP (mmol/l)					
l-Arg	973.0 ± 163.2	795.6 ± 237.0	773.2 ± 244.4	0.79	0.01
Placebo	966.7 ± 147.0	944.8 ± 365.7	1045.7 ± 282.9		

Interestingly to note that body weight, total cholesterol, HDL cholesterol, LDL cholesterol, and triglyceride levels similarly decreased in both groups at the end of the intervention study and at the end of the postintervention follow-up (Tables 1, 2). These data strongly support a correct adherence of life style modifications in both groups throughout the study as reported in Table 3. In fact, compared to baseline, physical activity was significantly increased and total energy dietary intake, daily fat, and carbohydrate intake were significantly decreased alike in both groups during the interventional study and during the postintervention follow-up period.

**Table 3:** ANCOVA analysis of dietary intake and physical activity in 35 participants in the L-Arg group and 21 participants in the Placebo group that remained non-diabetic at the end of the postintervention follow-up (mean ± SD)

Variable	Baseline	Intervention study	Postintervention follow-up	Time *p* value	Time for treatment *p* value
Dietary intake					
Energy (kJ)					
l-Arg	7942 ± 1367	7222 ± 1697	7390 ± 1907	0.05	0.55
Placebo	7911 ± 1577	7193 ± 1823	7172 ± 1355		
Fat (g)					
l-Arg	65.5 ± 16.9	62.7 ± 18.6	64.5 ± 19.0	0.39	0.15
Placebo	63.9 ± 15.3	66.8 ± 20.8	59.1 ± 20.8		
Saturated fat (g)					
l-Arg	21.5 ± 10.7	18.0 ± 7.2	18.49 ± 6.2	0.01	0.12
Placebo	21.1 ± 9.6	19.5 ± 9.1	17.3 ± 9.1		
Monounsaturated fatty acids (g)					
l-Arg	25.5 ± 8.5	24.9 ± 9.9	21.2 ± 7.9	0.05	0.32
Placebo	23.4 ± 7.6	25.5 ± 13.2	21.4 ± 13.0		
Polyunsaturated fatty acids (g)					
l-Arg	10.0 ± 5.0	14.6 ± 9.9	19.0 ± 9.6	0.01	0.75
Placebo	11.2 ± 6.3	16.7 ± 7.9	14.5 ± 9.0		
Cholesterol (g)					
l-Arg	360.7 ± 43.7	267.7 ± 79.6	275.7 ± 72.4	0.01	0.97
Placebo	324.8 ± 58.8	288.7 ± 100.1	239.3 ± 54.3		
Carbohydrates (g)					
l-Arg	254.4 ± 51.6	213.9 ± 71.0	209.9 ± 67.0	0.01	0.61
Placebo	264.6 ± 70.1	200.7 ± 73.7	212.2 ± 66.4		
Protein (g)					
l-Arg	75.9 ± 20.4	76.2 ± 22.4	77.7 ± 19.4	0.12	0.18
Placebo	75.8 ± 24.1	75.0 ± 20.2	77.5 ± 18.4		
l-Arginine (g)					
L-Arg	3.5 ± 1.0	4.0 ± 1.6	4.0 ± 2.0	0.08	0.45
Placebo	3.6 ± 1.2	4.0 ± 1.7	4.1 ± 1.5		
Total fibre (g)					
l-Arg	25.9 ± 9.3	20.1 ± 13.0	18.4 ± 5.3	0.07	0.57
Placebo	26.0 ± 8.7	19.7 ± 7.4	17.8 ± 6.1		
Physical activity					
Total activity (h/week)					
l-Arg	1.7 ± 2.7	2.9 ± 3.1	2.7 ± 3.2	0.01	0.16
Placebo	1.9 ± 1.9	3.1 ± 2.2	3.4 ± 2.6		

### Exploring a possible legacy effect of l-arginine on insulin secretion and insulin sensitivity, and on endothelial and oxidative stress

We measured the change difference of insulin secretion and insulin sensitivity, oxidative stress, and endothelial function indices during the postintervention period compared to results of each variable obtained at the end of the intervention study to evaluate a further effect of arginine after its discontinuation. As reported in Fig. 4b, there was a progressive increase in proinsulin difference in both groups with a more pronounced effect in the placebo group starting from 78 months after the end of the intervention study and thereafter. At the end of the study, change in proinsulin levels was threefold higher in placebo group than in l-arginine group (l-arginine group: 3.47 ± 1.52 vs. placebo group: 10.12 ± 4.40 pmol/l, *p* < 0.05). Moreover, changes in proinsulin/c-peptide ratio were 6.5-fold higher in the placebo group then in l-arginine group (l-arginine group: 1.34 ± 2.24 vs. placebo group: 8.77 ± 3.45 × 10^−3^, *p* < 0.05). Changes in IGI/HOMA-IR ratio were negatives in the placebo group starting from 72 months after the end of the intervention study and thereafter suggesting an important reduction in the disposition index after placebo treatment. Conversely, changes in IGI/HOMA-IR ratio remained unchanged suggesting a preservation of this activity after l-arginine therapy (at 108 months: l-arginine group: 1.66 ± 3.52 vs. placebo group: −57.02 ± 14.48, *p* < 0.05).

Finally, the analysis of changes during this period for AOPP indicates a further beneficial effect of l-arginine on oxidative stress that remained active for at least 48 months after the end of the intervention study (at 48 months: − 64.4 ± 300.9 vs. 122.1 ± 193.3 μmol/l, *p* < 0.05; Fig. 5b). Conversely, similar differences were reported for ADMA and EPC-CFU in the two groups of subjects (Fig. 5b).

All these data may suggest that l-arginine supplementation determined a legacy effect on preservation of β-cell function associated with a reduction in oxidative stress.

### Possible indices of prediction of diabetes throughout the 108 months of observation in this cohort of subjects

To evaluate whether it is possible to predict subjects at very high risk to become diabetics, we retrospectively analyzed the results dividing the subjects in three groups according to the time to disease incidence, independently of study treatment: the first group consisted in subjects that became diabetics during the intervention study, the second group consisted in subjects that became diabetics during the postintervention follow-up and the third group consisted in the subjects that remained free of diabetes during the postintervention follow-up. At baseline, the measurement of the proinsulin/c-peptide ratio showed that there was a staircase decrease of this index in the three groups with the highest ratio in the first group and the lowest ratio in the third group (38.3 ± 14.1, 36.3 ± −18.4, and 30.2 ± 17.8 × 10^−3^, *p* < 0.05 for trend). Interestingly, the measurement of proinsulin/c-peptide ratio at the end of the intervention period indicated that this variable was higher in the group of subjects that became diabetics during the post intervention follow-up period than in subjects that remained free of diabetes during the same period (30.4 ± 31.8 vs. 17.2 ± 25.9 × 10^−3^, *p* < 0.05). It is important to underline that in the group of subjects that remained free of diabetes throughout the postintervention period, proinsulin/c-peptide ratio at the end of the 108 months of follow-up was 11.2 ± 13.8 and 47.3 ± 46.6 × 10^−3^ (*p* < 0.01) in l-arginine and in placebo groups, respectively, suggesting a very important protective effect of l-arginine on β-cell function.

## Discussion

The results of the present study demonstrated the beneficial effects of l-arginine on glucose tolerance, insulin sensitivity, and insulin secretion, reducing the progression to T2DM. These effects were maintained for at least 90 months after the end of intervention study and could be mediated in some extent by l-arginine-induced reduction in oxidative stress.

In particular, in the l-arginine group, we found a decrease of 20% of total subjects with diabetes when compared to the placebo group during the 9-year period. Previously, a similar result was achieved only in intensive lifestyle modification program after 3 years. On the contrary, in the placebo group, the results obtained from the present study were very similar to results found in the Diabetes Prevention Program Outcome Study after 10-year follow-up [33]. In that study, after 10-year follow-up, in the control group diabetes incidence reached almost 65% of subjects.

l-Arginine is the substrate for production of NO and its second messenger cGMP, so it is expected that part of beneficial effects of l-arginine on insulin sensitivity is related to an amelioration of endothelial dysfunction. In fact, it has been previously demonstrated that l-arginine potentiates insulin-mediated glucose uptake by increasing blood flow with a mechanism related to insulin signaling [15–20].

In the present study, we found possible lasting effects of l-arginine on oxidative stress. However, in the present study, only one marker of oxidative stress was used and conclusive conclusions need to be confirmed by other oxidative stress markers. On the other hand, AOPPs may be a potential inducer of cellular inflammation in vivo under certain pathophysiological circumstances [23]. More recently, Liang et al. found that AOPPs directly promote NADPH oxidase-dependent β-cell destruction and dysfunction [7]. The l-arginine efficacy on oxidative stress may be attributed either through l-arginine/nitric oxide pathways [34] or by a direct antioxidant effect that is due to the alpha-amino group, a chemical moiety different from that necessary for NO generation. By acting as an antioxidant, l-arginine may scavenge O_2_- and thereby prevent eNOS-mediated O_2_ production in an uncoupled status [35]. Moreover, l-arginine is well known to regulate endocrine pancreatic functions [36] and dose-dependently prevents cytokine-induced β-cell apoptosis due to its conversion to l-glutamate, which enhances antioxidant defenses [37]. The unchanged proinsulin levels in l-arginine group suggested that l-arginine could improve insulin release granting sufficient insulin production and amelioration of β-cell machinery for some years after interruption of the treatment. The positive effects on proinsulin/c-peptide also corroborate our results [27, 38]. These data are in line with the previous animal and in vitro evidences that pretreatment with l-arginine and/or sodium nitroprusside (a donor of nitric oxide) has a protective action against alloxan-induced β-cell damage [2, 21]. In the same experimental model of alloxan-induced β-cell damage, l-arginine induced an increase of insulin immunopositivity in endocrine tissue of diabetic pancreas exposed to alloxan, suggesting the presence of β-cells neogenesis [22]. Our results also support the hypothesis that proinsulin/c-peptide ratio is a prognostic index of beta-cell dysfunction, as elegantly demonstrated by Zethelius et al. [39] and that l-arginine supplementation is able to improve β-cell function in the early steps of the evolution toward the diagnosis of type 2 diabetes.

On the other hand, other hypothesis might be drawn regarding the mechanisms by which l-arginine treatment could induce a long-lasting improvement in insulin secretion. In diabetes and prediabetes, among many molecular mechanisms involved, there is a reduced NO bioavailability, which is linked with altered intra-cellular signaling. The key role is played by the insulin-stimulated PI3K/Akt/eNOS signaling and pathway, that are impaired in insulin resistance, so in prediabetes [6, 7]. It could be possible an increased Akt and eNOS gene expression during l-arginine treatment with a potential long-lasting beneficial role of l-arginine on insulin sensitivity and secretion [40] and endothelial function [17–20]. The results of improvement in EPCs number and ADMA levels in subjects receiving l-arginine are, in our opinion, of particular interest, suggesting that l-arginine is able to enhance the expression levels of genes involved in metabolic and endothelial function. It is well known that individuals with T2DM or prediabetes have reduced levels of circulating EPCs and increased ADMA level, which correlated with disease severity [41–46].

It is also interesting to note that this is the first study able to demonstrate a “metabolic memory” in the prevention of T2DM. Previously, the concept of a metabolic memory was considered only for the ability to decrease the incidence of diabetes complications as demonstrated by the Diabetes Control and Complications Trial (DCCT) published in 1993, and his follow-up, the Epidemiology of Diabetes Interventions and Complications (EDIC) Study [47]. Other trials with type 2 diabetic patients have also found that the benefits of intensive glycemic control lasted long time after cessation of intervention [48, 49]. Since, in the long-term study, l-arginine had no adverse events, it is possible to suggest the use of l-arginine in subjects with glucose intolerance and cardiovascular disease, to prevent the development of diabetes.

The main limitation of this study is the relative small number of subjects studied and further studies with a large number of subjects are needed. However, the evidence that, in term of lifestyle, the subjects had equal values between both groups (compared to baseline, body weight, lipid profile, and physical activity significantly improved and energy dietary intake significantly decreased in both groups), and the accuracy in the methodology of the evaluation of glucose tolerance, insulin sensitivity, and insulin secretion are the major strengths of this study and support our conclusions. Moreover, the food intake was established through a 3-day recall diary. Furthermore, also the previous research in this area has shown that the adult diet is quite stable over time. [50].

## Conclusions

The results of the present study may suggest that the administration of l-arginine could delay the development of T2DM for a long period. This hypothesis is supported by the evidence that subjects submitted to l-arginine therapy had a persistent improvement in insulin sensitivity and insulin secretion, compared with placebo during a very long follow-up period.

Moreover, as far as we know, the present study is the first one able to show that a nutritional supplementation treatment can determine an additive effect on lifestyle intervention on the incidence of T2DM during a long follow-up period, despite the fact that both groups of subjects maintained control of lifestyle factors. We could also demonstrate a decrease in proinsulin concentrations by l-arginine. Thus, the fact that l-arginine could be considered a very new and interesting drug of modern prediabetes treatment relates to the antioxidant and “beta-cell protective” effects.
